# Supported by science?: what canadian naturopaths advertise to the public

**DOI:** 10.1186/1710-1492-7-14

**Published:** 2011-09-15

**Authors:** Timothy Caulfield, Christen Rachul

**Affiliations:** 1 Health Law and Science Policy Group, University of Alberta, Edmonton, Canada; 2 Faculty of Law, University of Alberta, Edmonton, Canada

## Abstract

**Background:**

The increasing popularity of complementary and alternative medicines in Canada has led to regulatory reforms in Ontario and British Columbia. Yet the evidence for efficacy of these therapies is still a source of debate. Those who are supportive of naturopathic medicine often support the field by claiming that the naturopathic treatments are supported by science and scientific research.

**Methods:**

To compare provinces that are regulated and unregulated, we examined the websites of 53 naturopathic clinics in Alberta and British Columbia to gain a sense of the degree to which the services advertised by naturopaths are science based.

**Results:**

There were very few differences between the provinces in terms of the types of services offered and conditions treated. Many of the most common treatments--such as homeopathy, chelation and colon cleanses--are viewed by the scientific community to be of questionable value and have no scientific evidence of efficacy beyond placebo.

**Conclusions:**

A review of the therapies advertised on the websites of clinics offering naturopathic treatments does not support the proposition that naturopathic medicine is a science and evidence-based practice.

## 

In recent years, naturopathic medicine has gained popularity as a form of primary care. In fact, a recent Canadian study found that 13% of children with asthma used complementary and alternative medicine (CAM) to treat their asthma [1]. With this increase in popularity, a number of provinces have granted more official status to these and other CAM practitioners [2], including regulatory reform in Ontario and British Columbia (BC) that expanded naturopaths' scope of practice to include allergy testing and treatment, as well as new prescribing rights, among other things.

However, the growth in naturopathic medicine has not been welcomed by all. Many critics contend that naturopathy treatments are not supported by scientific evidence [3]. Others have gone so far as to suggest that naturopathy is "no more based on science than astrology" and that it is just "a hodgepodge of alternative healthcare practices that are said to boost the body's natural healing powers" [4]. The lack of scientific evidence in naturopathic practices has been a particular concern for health professionals in the context of allergy and asthma [5,6].

In response, a variety of commentators, including many involved in the profession, have stated that the remedies provided by naturopathic practitioners are supported by solid research. Indeed, claims of scientific rigor have emerged as central to the push for expanded scopes of practice and mainstream acceptance. For example, the British Columbia Naturopathic Association (BCNA) claims that, "[t]here is a wealth of research, both controlled and double-blind clinical studies, showing the scientific basis and validity of naturopathic protocols" http://www.bcna.ca/files_3/naturophatic.php. The website for the Association of Accredited Naturopathic Medical Colleges similarly states that, "diagnoses and therapeutics are science based and increasingly evidence based" http://www.aanmc.org/careers/todays-naturopathic-doctors.php.

But is this true? Are the treatments that are offered by naturopaths science based? In order to explore this question, we examined the websites for naturopathic clinics in Alberta, where naturopaths are not regulated, and British Columbia, where they are. The sample, which was obtained using the Alberta Association of Naturopathic Practitioner's registration list and the BC Naturopathic Association ND list, totaled 53 websites for each province. The goal of this survey was to gain a sense of the degree to which the services advertised by naturopaths are, in a broad sense, science based.

What did we find? A list of largely scientifically unsupportable treatments and services [See Table 1]. In Alberta, for example, homeopathy was the most common treatment advertised on the websites (94% of websites note this treatment) and the third most common in BC (79%). Within the non-CAM scientific community, homeopathy has long been viewed as a sham [7]. Recently, this skeptical view has gained more traction in the public and policy spheres. In February of 2010, for example, the British Parliament's Science and Technology Committee released a summary of the evidence on homeopathy [8]. The report, which involved consultation with experts and an analysis of the available clinical evidence, concluded that homeopathy does not work, at least no better than placebo, and that the foundational principle of like-cures-like is "theoretically weak." More important, at least in relation to the claim that the treatment is science based, was the conclusion that the very idea behind homeopathy - that "ultra-dilutions can maintain an imprint of substances previously dissolved in them" - is "scientifically implausible" [8]. In other words, homeopathy does not work and there is no scientific reason to think that it could work.

**Table 1:** Most common treatments, testing, and treated conditions advertised on Alberta and BC naturopath clinics' websites.

	Alberta (53 clinic websites)	British Columbia (53 clinic websites*)
**Treatments**	**53 Total** **Top ten:**	**56 Total** **Top ten:**
	1. Homeopathy (50, 94%)	1. Botanical Medicine (45, 85%)
	2. Clinical Nutrition (49, 93%)	2. Clinical Nutrition (44, 83%)
	3. Botanical Medicine (47, 89%)	3. Homeopathy (42, 79%)
	4. Acupuncture and Traditional Chinese Medicine (44, 83%)	4. Acupuncture and Traditional Chinese Medicine (40, 76%)
	5. Lifestyle Counseling (38, 72%)	5. Lifestyle Counseling (31, 59%)
	6. Detoxification or Biotherapeutic Drainage (27, 51%)	6. Detoxification or Biotherapeutic Drainage (24, 45%)
	7. Massage (26, 49%)	7. IV Therapies (22, 41%)
	8. Hydrotherapy (24, 45%)	8. Bowen Technique (19, 36%)
	9. Chiropractics and/or Cranial Sacral Manipulation (23, 43%)	9. Chiropractics and/or Cranial Sacral Manipulation (17, 32%)
	10. IV Therapies (20, 38%)	10. Neural Therapy (15, 28%)

**Testing or Lab Analysis**	**43 Total** **Top ten:**	**42 Total** **Top ten:**
	1. Allergy Testing (24, 45%)	1. Heavy Metal Testing/Chelation Therapy (23, 43%)Hormone Testing (23, 43%)
	2. Hormone Testing (23, 43%)	2. Allergy Testing (22, 42%)
	3. Heavy Metal Testing/Chelation Therapy (20, 38%)	3. Blood Work (14, 26%)
	4. Urinalysis (14, 26%)	4. VEGA (Electro-dermal testing) (13, 25%)
	5. Bio Impedence Analysis (10, 19%) Hair Analysis (10, 19%)	5. Urinalysis (12, 23%) Hair Analysis (12, 23%)
	6. Adrenal Testing (9, 17%) Blood Work (9, 17%)	6. Blood Typing (11, 21%)
	7. VEGA (Electro-dermal testing) (6, 11%) Live Blood Cell Analysis (6, 11%) Thyroid Testing (6, 11%) Bowel Toxicity (6, 11%)	7. Gynecological Exam (10, 19%) Annual Physical Exam (10, 19%)
	8. Breast Exams (5, 9%) Blood Typing (5, 9%) Gynecological Exam (5, 9%) Neurotransmitter Testing (5, 9%)	8. Stool Analysis (9, 17%)
	9. Annual Physical Exam (4, 7.5%) Genomic Testing (4, 7.5%)	9. Parasite Testing (7, 13%)
	10. Estrogen Metabolism Testing (3, 6%) Zinc Tally Testing (3, 6%) Body Composition Testing (3, 6%) Parasite Testing (3, 6%)	10. Thyroid Testing (6, 11%) Adrenal Testing (6, 11%) Bio Impedence Analysis (6, 11%)

Ailments Treated**	**40 Total** **Top ten:**	**43 Total** **Top ten:**
	1. Allergies (32, 60%)	1. Allergies (25, 47%)
	2. Digestive Disorders (28, 53%)	2. Women's Health (24, 45%)
	3. Pain (Chronic and acute) (26, 49%) Women's Health (26, 49%)	3. Digestive Disorders (19, 36%)
	4. Fatigue, Low Energy (25, 47%) Weight Management/Obesity (25, 47%)	4. Cardiovascular Issues (18, 34%)
	5. Fertility Issues (24, 45%) Cardiovascular Issues (24, 45%)	5. Fatigue, Low Energy (17, 32%) Weight Management/Obesity (17, 32%)
	6. Skin Disorders (22, 42%) Headaches/Migraines (22, 42%)	6. Diabetes (14, 26%)
	7. Diabetes (21, 40%) Sport Injuries (21, 40%)	7. Pain (Chronic and Acute) (13, 25%) Skin Disorders (13, 25%) Arthritis (13, 25%) Cold and Flu (13, 25%)
	8. Autoimmune Diseases (20, 38%) Chronic Illnesses (20, 38%) Arthritis (20, 38%)	8. Autoimmune Diseases (12, 23%) Depression/Anxiety (12, 23%) Fertility Issues (12, 23%) Menopause (12, 23%) Sport Injuries (12, 23%) Fibromyalgia (12, 23%)
	9. Menopause (19, 36%) Pre- and Post-natal Health (19, 36%) Fibromyalgia (19, 36%)	9. Cancer (11, 21%) Stress (11, 21%)
	10. Cancer (18, 34%)	10. Thyroid Problems (10, 19%) Headaches/Migraines (10, 19%)

* There are 191 clinic websites listed for BC. We chose a random sample of 53 clinics to match the number of Alberta clinic websites we examined.

**This includes specific ailments or conditions that were named, but many websites finish their lists with: "and many more" or "This is only a sampling of conditions treated at our centre", or some claim, "Naturopathic Physicians work with virtually all acute and chronic conditions".

Many of the other services and tests advertised to the public by naturopaths, such as colon hydrotherapy, detoxification and hair analysis are, from a scientific perspective, equally spurious (the American Medical Association, for example, has stated that hair analysis is an unproven diagnostic technique and has the potential to lead to "health care fraud" [9]). And a large percentage of the clinics also offer heavy metal testing for the purposes of chelation therapy. Unless these clinics are providing this service for the treatment of lead poisoning, the only use of chelation therapy for which there is evidence of efficacy, than this treatment option is also not supported by scientific evidence [10].

While naturopaths are undoubtedly providing other services that have a stronger evidence base (it is hard to argue with the value of sound nutrition advice), this review of what they advertise as their core services paints a picture of a profession that has embraced practices that are remarkably unscientific. Despite this reality, many of the clinic websites also claim that their services are "evidence" and/or "science" based. One website declares that their therapies are "derived largely from scientific research conducted by the same universities, laboratories and medical schools that do the research on drugs and surgery" http://www.demontecentre.com. Another asserts that "[n]aturopathic medicine is patient treatment in the best possible way; personal, individual, caring as well as evidence based and scientific." http://www.abbottnaturopathic.com/

To be fair, some may argue that the evidence surrounding practices like homeopathy, hair analysis, colon cleansing and chelation is not as settled as we claim. It is true that both a lack of funding for CAM research (e.g., few patentable products means less industry support for clinical trials) and methodological challenges [11] means there is often a modest amount of empirically robust data to work with. But, as noted above, for many of the core naturopathic treatments, clinical studies and thorough scientific critiques of the foundational rationales do exist [12-17]. Even the most generous interpretation of this literature would not support a characterization of "science based," if this phrase is meant to imply the therapies have been shown to be efficacious using traditional scientific methodologies. These services are, at best, on the margins of scientific legitimacy [e.g., [8]].

There is no doubt that other healthcare professionals, including physicians, provide therapies that are not supported by solid empirical evidence. And a wide range of social forces, including a pervasive industry bias, often distorts the evidence that is used [18]. These are serious problems, for sure. But the evidence issues of the medical profession cannot, obviously, stand as a justification for not using evidence in the context of naturopaths. All healthcare options should, as much as possible, be informed by good science [19]. To this end, the medical profession has a stated commitment to evidence-based practice and is taking steps to deal with the deficits in the production and use of evidence. As evidence improves, new practices are adopted and those shown to be ineffective are dropped.

If the naturopathic medicine were truly "science based", as so often claimed by the advocates of the field, would they still be providing homeopathy as one of their core treatments? Would chelation and colon cleanses be marketed on so many of the clinics' websites?

Patients should have the option to access a wide range of healthcare practitioners. But this choice should be as informed as possible. As should the policy debates associated with the regulation of naturopaths' scope of practice. This is particularly so given the types of conditions they seek to address. While one could argue that homeopathic treatment for the common cold is harmless, treatment for conditions such as allergies, cardiovascular problems, fertility issues and cancer raises serious ethical and legal questions. It is misleading to imply that the core services provided by naturopaths - as disclosed on clinic websites - are based on sound scientific evidence or, at least, that there is a scientific consensus about their efficacy. According to clinic websites, allergy testing and treatment are among the most common services provided in both BC and Alberta (e.g., VEGA testing or homeopathic allergy desensitization), yet there is no scientific consensus for the efficacy of naturopathic methods for testing or treatment [5,6]. It can also be argued that allergy testing performed by naturopaths are both expensive and potentially misleading, which could result in inappropriate dietary modifications [e.g., [20]]. If the naturopathic profession wishes to present itself as science based, the treatments offered by naturopath clinics should reflect these claims.

As noted in a recent speech by the President of the American Association of Naturopathic Physicians, "if our profession is to be taken seriously by the larger world of medicine, we must speak in a language that everyone can understand and appreciate. That language is the language of science." [21]. We couldn't agree more. But the profession should not just use the language of science, it must embrace and act on the conclusions of scientific inquiry.

## Competing interests

The authors declare that they have no competing interests.

## Authors' contributions

TC designed the study, CR collected the data, both authors contributed to drafts of the manuscript and approved the final manuscript.
